# A Facile, Sustainable One-Pot Synthesis of the Spiro-Dimers of α-Tocopheramine and Its *N*-Methyl Derivative

**DOI:** 10.3390/molecules30061269

**Published:** 2025-03-12

**Authors:** Anjan Patel, Thomas Rosenau

**Affiliations:** 1 Department of Chemistry, Institute of Chemistry of Renewable Resources, University of Natural Resources and Life Sciences, Vienna (BOKU), Konrad-Lorenz-Straße 24, 3430 Tulln, Austria; 2 Laboratory of Natural Materials Technology, Faculty of Science and Engineering, Åbo Akademi University, Porthansgatan 3, FI-20500 Åbo/Turku, Finland

**Keywords:** cellulose, fiber spinning, chromophore, oxidation, spiro-dimerization, tocopheramine

## Abstract

α-Tocopheramine and *N*-methyl-α-tocopheramine are used as stabilizers in the spinning of cellulosic fibers from solutions in 1,3-dialkylimidazolium ionic liquids. Upon aging, they form chromophoric degradation products, among which the spiro-dimers are major components. These compounds have proved to be otherwise inaccessible so far, although they are urgently needed as chromatographic standards in spinning baths and fiber analysis. In this work, the spiro-dimers of α-tocopheramine and *N*-methyl-α-tocopheramine have been synthesized. Extensive optimization of reaction conditions, solvents and oxidants resulted in a sustainable one-pot protocol that provided quantitative yields without the need for product purification and with the easy recycling of the solvent.

## 1. Introduction

**Scheme 1:**
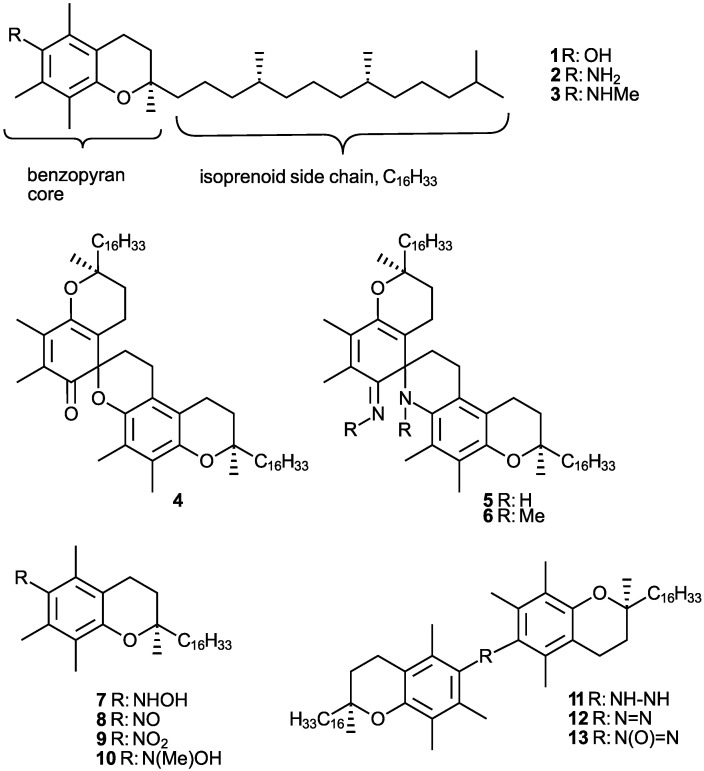
Chemical structures of parent compound α-tocopherol (**1**), α-tocopheramine (**2**) and its *N*-methyl derivative (**3**), their spiro-dimers (**4**–**6**) oxidation products (**7**–**13**).

Tocopheramines are aza-derivatives of tocopherols, with *α*-tocopherol (**1**) being the main component of vitamin E [1]. The phenolic OH group in *α*-tocopherol is replaced by an amino group (-NH_2_) in α-tocopheramine (**2**), but also the *N*-methyl derivative (**3**) and *N*,*N*-dimethyl derivative (**4**) are rather common [2,3] (Scheme 1). The tocopheramines have recently achieved larger-scale applications as stabilizers in the spinning of cellulosic fibers from solutions in 1,3-dialkylimidazolium ionic liquids, where they reduce the autoxidative degradation of both cellulose and solvent and reduce the discoloration of the spinning dope and the resulting fibers. In this application, they have been superior to their phenolic tocopherol counterpart [4,5]. In addition to its effectivity in ionic liquid solutions of cellulose, it is also an effective stabilizer of cellulose solutions in *N*-methylmorpholine *N*-oxide, which is the solvent used in the industrial Lyocell fiber-making process [6]. 

Apart from these larger-scale applications, tocopheramines have been studied with regard to several applications in the food and medicinal fields. Being fully biocompatible and non-toxic, similar to the tocopherols [2,7], tocopheramines have been tested as food [8] and feed additives [9], polymer stabilizers [10] and surfactants [11]. As lipophilic radical scavengers in cell models, they even surpassed their better-known phenolic counterparts in several cases [12,13]; their anticancer and proapoptotic activities [14,15], for instance, are superior to the well-studied α-tocopheryl acetate and succinate [16,17,18,19]. 

Tocopheramines have been studied with regard to their degradation mechanisms and products. These aging products are mainly *N*-oxidized compounds with one benzopyran moiety, such as the hydroxylamino (**7**), *N*-methyl-*N*-hydroxy (**10**), nitroso (**8**) or nitro (**9**) derivatives [20,21], or *N*-oxidized dimeric compounds (having two benzopyran moieties) [22], such as the hydrazine (**11**), azo (**12**) or azoxy (**13**) derivatives of tocopheramine (Scheme 1). High-yield syntheses for these compounds, without the need to separate structurally very similar derivatives, have been developed so that standards are available to assist byproduct identification in different application fields [19,20,21]. 

Upon the usage of tocopheramines as stabilizers in fiber making, spiro-dimeric compounds, which are the analogues of the well-known spiro-dimer derived from α-toco-pherol (**4**) [23,24], have been identified as novel degradation products of tocopheramines: the spiro-dimer of α-tocopheramine (**5**) and the spiro-dimer of *N*-methyl-α-tocopheramine (**6**) are even the dominant degradation products besides minor amounts of *N*-oxidized derivatives, see Scheme 1. While the spiro-dimer derived from α-tocopherol (**4**) is one of the most common derivatives in vitamin E chemistry and can readily be synthesized in quantitative yields by the two-electron oxidation of **1** in non-aqueous media, the aza-analogues **5** and **6,** derived from tocopheramines, cannot be prepared in a similar way, since common oxidation protocols produce only the *N*-oxidized derivatives, but not the spiro-compounds. The isolation from fibers stabilized by tocopheramine is not a viable option either, since the obtainable amounts are far too low to meet the needs of the standard compounds used in chromatography and process optimization. Additionally, isolation in the pure form has proven to be quite challenging. Thus, there was the need to provide the spiro-dimers (**5**,**6**) as neat compounds to be used as standards in chromato-graphy and quality control—if possible synthesized according to procedures that comply with green chemistry principles [25,26]— which could provide the compounds in a gram scale and without the need for extensive chromatographic work-up so that the protocol would also be usable in industrial quality assurance labs specialized in synthesis. This study describes the developed synthesis approach, its optimization and the comprehensive analytical characterization of the target compounds. 

## 2. Results and Discussion

**Scheme 2:**
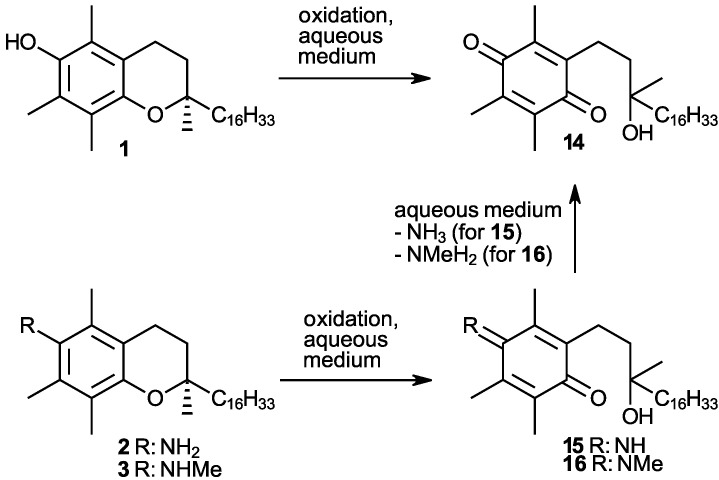
Oxidation of α-tocopherol (**1**) and α-tocopheramines (**2**,**3**) to *para*-tocopherylquinone (**14**) in aqueous media.

Similar to the oxidation of α-tocopherol (**1**) in an aqueous medium [27,28], the oxidation of α-tocopheramine (**2**) and *N*-methyl-α-tocopheramine (**3**) in aqueous media produced α-tocopherylquinone (**14**) as the main product. This compound is formed via the corresponding *para*-iminoquinones (**15**,**16**) which are immediately hydrolyzed in aqueous media to the corresponding *para*-quinone **14**, see Scheme 2. In general, in binary solvent systems containing more than a 20 vol% of water, α-tocopherylquinone (**14**) dominated by far, with yields above 60%. The *N*-methyl-*N*-hydroxy derivative (**10**) was another byproduct formed from *N*-methyl-α-tocopheramine (**2**), while the nitro derivative (**9**) and azo derivative (**12**) were the byproducts from α-tocopheramine. Working in a non-aqueous medium changed the product distribution in a way that the formation of the *N*-oxidized compounds was boosted at the expense of α-tocopherylquinone (**14**), which was only found in the presence of water. In a preliminary screening, 14 different oxidants were tested in common organic solvents, such as toluene, dioxane, THF, chloroform, cyrene, DMF and DMSO. They all achieved the full consumption of the α-tocopheramines, but provided only *N*-oxidized products, not the corresponding spiro-dimers (**5**,**6**). This was a fundamental difference with regard to the oxidation chemistry of α-tocopherol, in which the corresponding spiro-dimer (**4**) is known to be the dominating oxidation product in non-aqueous media, largely independent of the solvent and oxidant [23,24]. Only with toluene as the solvent and DDQ as the oxidant, did 4% of the spiro-dimer (**5**) form from α-tocopheramine (**2**), in addition to a mixture of the nitro derivative (**9**, 44%) and azo derivative (**12**, 52%).

**Scheme 3:**
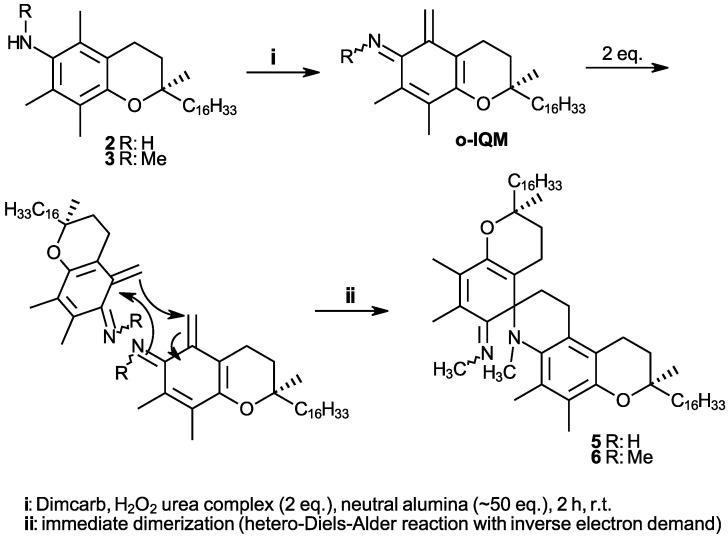
Oxidation of α-tocopheramines (**2**,**3**) to the corresponding spiro-dimers (**5**,**6**): mechanism and optimized reaction conditions.

In vitamin E chemistry, the oxidative formation of the *ortho*-quinone methide is a fundamental process. This intermediate undergoes immediate dimerization—in a hetero-Diels–Alder reaction with inverse electron demand (IEDDA)—to the corresponding spiro-dimer [1,29]. The regioselectivity of the *ortho*-quinone methide formation, i.e., the exclusive involvement of the C-5a methyl group instead of the C-7a alternative, has been explained by the theory of strain-induced bond localization (SIBL) [30]. For the case of the formation of the tocopheramine spiro-dimers, we assumed a mechanism similar to that of the α-tocopherol counterpart: a two-electron oxidation to the corresponding *ortho*-iminoquinone methide (**o-IQM**) with subsequent hetero-Diels–Alder dimerization, see Scheme 3. However, either the initial oxidation reaction or the subsequent dimerization process (or both) was rather disfavored in the tocopheramine case. Evidently, the amino function allowed for more alternative pathways—and thus byproducts—than the phenolic OH group in the tocopherol realm. We thus had to look for ways to enhance the *ortho*-iminoquinone methide formation over *N*-oxidation in order to obtain the targeted tocopheramine spiro-dimers (**5**,**6**) in at least moderate yields. 

It should be noted at this point that the tertiary *N,N*-dimethyl-α-tocopheramine cannot form the corresponding *ortho*-quinone methide, and is thus unable to form a corresponding spiro-compound. Nevertheless, the compound is important in tocopheramine chemistry as it can be easily and neatly demethylated into the *N*-monomethyl derivative (**3**) [31], which is difficult to obtain in similar purity by the (reductive) methylation of α-tocopheramine.

A first indication of the oxidation conditions favoring *ortho*-iminoquinone methide formation, was evidenced by the fact that the spiro-dimer of *N*-methyl-α-tocopheramine (**6**) was found in cellulose solutions studied by gel permeation chromatography, which uses the eluant *N,N*-dimethylacetamide/lithium chloride (DMAc/LiCl, 8 wt%). Evidently, atmospheric oxygen was able to oxidize *N*-methyl-α-tocopheramine (**3**) to the spiro-dimer (**6**) in this solvent system, which is a standard solvent in cellulose analysis, but is rather exotic as a solvent in synthesis. Spin trapping with EMPO derivatives [32] confirmed the presence of *N*-centered and *O*-centered radical species, which were not further studied. When using DMAc as a neat solvent with two equivalents of either of the four oxidants, NMMO, DDQ, HOCl (from NMMO/AuCl_3_ (cat.) [33] and Pb(OAc)_4_ or hydrogen peroxide (30%), no spiro-dimer was obtained at all. However, in DMAc containing the salt LiCl (8 wt%) the yields of spiro-dimer **5** in the case of the four oxidants were 35%, 36%, 24% and 48%, respectively. From this observation, we concluded that the presence of salts or ionic components was beneficial for *ortho*-iminoquinone methide formation, apparently by stabilizing the zwitterionic canonic form of this intermediate. While in these four cases the starting tocopheramine had been fully consumed, the nitro and azo derivative were the main products with DDQ and Pb(OAc)_4_, while 22% of *para*-tocopherylquinone was formed in the case of hydrogen peroxide (30%) as the oxidant, evidently involving the water added with the aqueous oxidant solution (and also the water resulting from its reduction). In the next step, we thus used the solid urea–H_2_O_2_ complex (two eq.) instead of aqueous hydrogen peroxide, which boosted the yield of the spiro-dimer **5** from α-tocopheramine (**2**) to 72%, and 65% for the spiro-dimer **6** from *N*-methyl-α-tocopheramine (**3**). The byproducts were once more the nitro (**9**) and the azo derivative (**12**) from **2**, and the hydroxylamine derivative **13** from **3**. Increasing the amount of oxidant used did not improve the yield, and neither did higher reaction temperatures (in 20 °C steps from 22 °C to 82 °C), which had no effect apart from a slight shift of the product ratio towards the *N,N*-coupled dimeric derivatives (azo (**9**) and hydrazine (**13**) compound from **2** and **3**, respectively).

Since high ionicity evidently had a positive effect, we changed to ionic liquids (ILs) as the reaction medium. The stabilizing effect of ionic—in particular zwitterionic—species on tocopherol-derived *ortho*-quinone methides has been described [34,35] and attributed to the stabilizing Coulomb interactions of the ions towards their zwitterionic (aromatic) resonance structure. The use of ionic liquids as solvents also seemed logical from the perspective that the spiro-dimers were formed as degradation products when cellulose solutions stabilized by the tocopheramines were spun from solutions in these solvents [36]. We used 1-ethyl-3-methylimidazolium (EMIm) and 1-butyl-3-methyl imidazolium (BMIm) ionic liquids, either as acetate or chloride, as the solvents, with either the urea–peroxide complex or 30% aqueous hydrogen peroxide as the oxidants. The outcome was quite encouraging (Table 1), with the yields of the chloride ILs being slightly but consistently higher than the acetate ILs. Two equivalents of oxidant gave a full conversion of the starting tocopheramine. Most importantly, no *N*-oxidized byproducts were observed, but just α-tocopherylquinone (**14**).

The formation of the *para*-quinone **14** evidently depended on the water content: the initially formed *para*-iminoquinones (**15**,**16**) are hydrolyzed to the *para*-quinone **14** by the water traces present, see Scheme 2. This explains the consistently higher yields of byproduct **14** when aqueous hydrogen peroxide, instead of the solid hydrogen peroxide-urea complex, was used as the oxidant. But even with the latter, the water traces generated upon H_2_O_2_ reduction were obviously sufficient to induce some *para*-quinone formation as a side reaction. The formation of *para*-quinone **14** was completely avoided by adding an excess (approx. 50 eq.) of neutral alumina (Brockmann grade 1) as an internal drying agent which immediately binds water once generated. It should be noted that neutral alumina produced better results than acidic alumina (some nitro derivative (**9**) formed) and alkaline alumina (dark red discoloration of the solution), and that Brockmann grades of high hygroscopicity (I or II) had to be used because less hygroscopic grades (III–V) did not efficiently prevent *para*-tocopherylquinone formation. Working in EMIM-Cl with 2 eq. of H_2_O_2_–urea complex as the oxidant and 50 eq. of neutral alumina, both spiro-dimers were quantitatively obtained from the corresponding starting amines, with all starting amines being consumed and no byproducts being detectable, see Table 1.

The solids used, excess oxidant and alumina, can be simply removed by filtration and the product, which is highly lipophilic due to the two isoprenoid C_16_ side chains, can be simply retrieved by extraction into a strongly apolar solvent, such as petrol ether or *n*-heptane. This enabled a fairly convenient work-up just by filtration and extraction, and eliminated the need of chromatographic purification. However, the complete removal of ionic liquid traces can be challenging. As 1,3-dialkylimidazolium IL traces might interfere with analytical detection and can even impair analytical hardware, such as GC capillaries or IC electrodes [37], this issue could not be disregarded. Therefore, as the last step of the optimization, we used another IL, dimethylammonium dimethylcarbamate (dimcarb), to replace the imidazolium ILs used so far. Dimcarb is formed by the two gases dimethylamine and carbon dioxide in the stoichiometric ratio 2:1, as a clear, colorless low-viscous liquid. In contrast to the imidazolium ILs, which cannot be distilled and are hard to purify and to dry after use, dimcarb can be “distilled” at 65 °C. This process is formally a distillation, albeit with a different underlying mechanism: dimcarb degrades into the two gaseous components which recombine at lower temperatures again to re-form the IL. Using dimcarb as the solvent, along with 2 eq. of H_2_O_2_–urea complex as the oxidant and 50 eq. of neutral alumina and followed by filtration to remove solids and heating to 70 °C to remove the dimcarb solvent, yields of the spiro-dimer **5** from α-tocopheramine (**2**) and spiro-dimer **6** from *N*-methyl-α-tocopheramine (**6**) were quantitative (Scheme 3). The compounds were obtained as colorless waxes without byproducts. Isolated yields after the chromatographic purification for samples of analytical purity were 93% and 90% for **5** and **6**, respectively.

When using dimcarb, some minute traces of chromophores that usually give the reaction solution a yellow color were absorbed to the alumina and thus removed together with the solids to produce colorless products, while in imidazolium ILs these chromophores remain dissolved so that the products appeared yellow in these cases. In addition to an even easier work-up—without theextraction step, just by filtration and solvent evaporation—this simultaneous chromophore removal is an additional advantage of using dimcarb over the imidazolium ionic liquids, although the yields for both solvents are comparable.

## 3. Materials and Methods

All chemicals were of the highest purity available and used without further purification. Bidistilled water was used for all aqueous solutions, extractions and washing steps. 1,4-Dioxane, *n*-heptane, ethyl acetate and toluene used in chromatography were distilled before use. The EMIm-Cl ionic liquid was obtained from Nippon Nyukazai Co., Ltd. (Tokyo, Japan).

TLC was performed using Merck silica gel 60 F254 precoated plates, and flash chromatography on Baker silica gel (40 µm particle size). All products were purified to homogeneity by TLC/GC analysis and satisfying elemental analysis data (± 0.3%). Elemental analyses were performed at the Microanalytical Laboratory of the University of Vienna. The melting points are corrected (benzophenone 48–49 °C and benzoic acid 122–123 °C) and determined on a Kofler-type micro hot stage. 

GC-MS/FID and UPC2-ESI-QTof-MS analyses were performed as previously described by Barbini et al. [38,39]. UV/Vis spectra were recorded on a LAMBDA 45 UV/Vis spectrophotometer (Perkin Elmer, Waltham, MA, USA): range of 400 to 700 nm, scanning speed 480 nm min^−1^, quartz glass cuvettes (l = 1.0 cm).

NMR analysis and identification of chromophores. All NMR spectra were recorded on a Bruker Avance II 400 (resonance frequencies 400.13 MHz for ^1^H and 100.63 MHz for ^13^C) equipped with a 5 mm N2-cooled cryoprobe head (Prodigy) with z-gradients at room temperature with standard Bruker pulse programs. The sample was dissolved in 0.6 mL of CDCl_3_ (99.9% D). Chemical shifts are given in ppm, referenced to residual solvent signals. ^1^H NMR data were collected with 32k complex data points and apodized with a Gaussian window function (lb = −0.3 Hz and gb = 0.3 Hz) prior to Fourier transformation. ^13^C spectrum with WALTZ16 1H decoupling was acquired using 64k data points. Signal-to-noise enhancement was achieved by multiplication of the FID with an exponential window function (lb = 1 Hz). All two-dimensional experiments were performed with 1k × 256 data points, while the number of transients (2–16 scans) and the sweep widths were optimized individually. HSQC experiment was acquired using adiabatic pulse for inversion of ^13^C and GARP-sequence for broadband ^13^C-decoupling, optimized for ^1^*J*_(CH)_ = 145 Hz. For the NOESY spectra, a mixing time of 0.8 s was used. 

The nomenclature and atom numbering of tocopherols and chromanols as recommended by IUPAC [40,41] was used throughout. ^1^H and ^13^C NMR resonances of the isoprenoid side chain of tocopherols are only insignificantly influenced (Δ < 0.05 ppm) by modifications of the chroman ring [42,43], and are thus listed only once: 19.7 (C-4a’), 19.8 (C-8a’), 21.2 (C-2’), 22.7 (C-13’), 22.8 (C-12a’), 24.6 (C-6’), 24.8 (C-10’), 28.0 (C-12’), 32.6 (C-8’), 32.8 (C-4’), 37.3 (C-7’), 37.4 (C-9’), 37.5 (C-5’), 37.5 (C-3’), 39.3 (C-11’) and 39.9 (C-1’). In the spiro-dimeric compounds **5** and **6**, the aromatic moiety is conventionally numbered, while the spiro-keto moiety is indicated by “#”. The analytical data [3] and NMR data [44] for α-tocopheramine (**1**) were in agreement with the literature.

Synthesis of the α-tocopheramine spiro-dimer (**5**). α-Tocopheramine ([*R,R,R*] isomer, **2**, 0.430 g, 1.00 mmol) was dissolved in dimcarb (200 mL) under stirring at RT. Neutral aluminum oxide (Brockmann grade 1, 5.00 g, 49 mmol) and urea–hydrogen peroxide complex (0.188 g, 2 mmol) were mixed as solids and the fine powder added through a dropping funnel at 5 min. The mixture was stirred for 2 h. Solids were removed by filtration and washed with dimcarb (twice 100 mL). The dimcarb phases were combined and heated in a rotavaporator at 80 °C to afford **5** as a waxy, colorless solid (0.43 g, quant.). The synthesis was repeated several times to afford sufficient amounts of target compound. An aliquot of 1 mmol was purified by flash chromatography (*n*-heptane/toluene, *v/v* = 3:1, 95% yield). 

Spiro-dimer of α-tocopheramine (**5**). Colorless wax, m.p. 48–50 °C, UV λmax (toluene): 284 nm. ^1^H NMR: δ 2.62 (2H, m, 4-CH_2_), 2.45 (2H, m, 4#-CH_2_), 2.24 (3H, s, 5a-CH_3_), 2.16 (3H, s, 5a#-CH_3_), 2.05 (3H, s, 7a-CH_3_), 1.83 (3H, s, 7a#-CH_3_), 2.04 (3H, s, 8b-CH_3_), 1.96 (3H, s, 8b#-CH_3_), 1.60–1.64 (4H, m, 3-CH_2_ and 3#-CH_2_), 1.04–1.50 (m, 42H, 18×CH_2_ and 6×CH in two isoprenoid side chains), 1.22 (3H, s, 2a-CH_3_), 1.21 (3H, s, 2a#-CH_3_), 0.82–0.92 (24H, m, 4a-CH_3_, 8a-CH_3_, 12a-CH_3_, 13-CH_3_, 4a#-CH_3_, 8a#-CH_3_, 12a#-CH_3_, 13#-CH_3_). ^13^C NMR: δ 145.3 (C-8a), 142.4 (C-8a#), 122.9 (C-8), 122.5 (C-8#), 10.8 (C-8b), 123.0 (C-8b#), 122.8 (C-7), 125.4 (C-7#), 11.0 (C-7a), 10.6 (C-7a#), 140.3 (C-6), 154.9 (C-6#), 112.5 (C-5), 68.7 (C-5#), 18.9 (C-5a), 25.8 (C-5a), 116.2 (C-4a), 116.1 (C-4a#), 19.0 (C-4), 16.5 (C-4#), 30.4 (C-3#), 30.2 (C-3), 73.7 (C-2#), 75.0 (C-2) and 22.3 (C-2a, C-2a#). Isoprenoid side chains: see above. Microanalysis: calcd. for C_58_H_98_N_2_O_2_ (855.41): C 81.44, H 11.55 and N 3.27, found: C 81.30, H 11.54 and N 3.08.

Synthesis of the *N*-methyl-α-tocopheramine spiro-dimer (**6**). The synthesis followed the above protocol, using *N*-methyl-α-tocopheramine ([*R,R,R*] isomer, **3** (0.444 g, 1.00 mmol) as the starting material. Product **6** was obtained as a waxy, colorless solid (0.445 g, quant.). The synthesis was repeated several times to afford sufficient amounts of target compound. An aliquot of 1 mmol was purified by flash chromatography (*n*-heptane/toluene, *v/v* = 3:1, 92% yield).

Spiro-dimer of *N*-methyl-α-tocopheramine (**6**). Colorless wax, m.p. 42–43 °C, UV λmax (toluene): 278 nm. ^1^H NMR: δ 2.60 (2H, m, 4-CH_2_), 2.46 (2H, m, 4#-CH_2_), 2.36 (3H, s, 5a-CH_3_), 2.18 (3H, s, 5a#-CH_3_), 2.05 (3H, s, 7a-CH_3_), 1.84 (3H, s, 7a#-CH_3_), 2.01 (3H, s, 8b-CH_3_), 1.96 (3H, s, 8b#-CH_3_), 1.59–1.64 (4H, m, 3-CH_2_ and 3#-CH_2_), 1.06–1.54 (m, 42H, 18×CH_2_ and 6×CH in two isoprenoid side chains), 1.22 (3H, s, 2a-CH_3_), 1.21 (3H, s, 2a#-CH_3_), 0.82–0.92 (24H, m, 4a-CH3, 8a-CH_3_, 12a-CH_3_, 13-CH_3_, 4a#-CH_3_, 8a#-CH_3_, 12a#-CH_3_, 13#-CH_3_). _13_C NMR: δ 145.4 (C-8a), 142.4 (C-8a#), 122.8 (C-8), 122.6 (C-8#), 10.7 (C-8b), 12.9 (C-8b#), 123.2 (C-7), 125.8 (C-7#), 10.9 (C-7a), 10.4 (C-7a#), 138.6 (C-6), 151.3 (C-6#), 111.2 (C-5), 68.7 (C-5#), 18.8 (C-5a), 25.5 (C-5a), 116.2 (C-4a), 116.0 (C-4a#), 18.8 (C-4), 16.6 (C-4#), 30.2 (C-3, C-3#), 73.8 (C-2#), 75.1 (C-2), 22.3 (C-2a, C-2a#), 38.8 (CH_3_-N) and 42.4 (CH_3_-N=). Isoprenoid side chains: see above. Microanalysis calcd. for C_60_H_102_N_2_O_2_ (430.66): C 81.57, H 11.64 and N 3.17, found: C 81.48, H 11.82 and N 3.11.

## 4. Conclusions

The α-tocopheramine spiro-dimer (**5**) and *N*-methyl-α-tocopheramine spiro-dimer (**6**) are formed as byproducts when the parent amines are used to stabilize cellulose spinning dopes in imidazolium ionic liquids. The isolation in pure form from these spinning dopes is not possible and the synthesis according to common procedures, such as those applied in α-tocopherol (vitamin E) chemistry, failed. Thus, the need for analytical and chromatographic standards required the search for more specialized synthesis approaches, which at the same time should be facile enough to also be usable in industrial quality assurance labs. With the presented synthesis, the following requirement is met: the approach is a one-pot protocol, it involves only filtration and solvent evaporation as work-up steps and does not require chromatographic purification. Moreover, the approach complies with green chemistry principles by avoiding toxic or hazardous organic solvents—the solvent used is dimcarb, an ionic liquid adduct of the two gases carbon dioxide and dimethylamine which is reversible degraded at 60 °C, and the oxidant is hydrogen peroxide (in solid form as urea adduct), which reacts to water in this process. The only other auxiliary involved is aluminum oxide. The provision of common stabilizer degradation products as analytical standards has removed another obstacle to the production of cellulosic fibers by spinning from ionic liquids and stabilizing this system with natural antioxidants. This work might also provide an impetus to investigate the hitherto insufficiently studied chemistry of *ortho*-iminoquinone methides, in particular their Diels–Alder reactions. 

## Figures and Tables

**Table 1:** Oxidation of α-tocopheramines (**2**,**3**) to the corresponding spiro-dimers (**5**,**6**) and byproduct formation depending on reaction parameters.

Conditions (Solvent, Oxidant and Auxiliaries) ^1^	Main Product (Yield%) ^2^	Byproduct 14 (Yield%) ^2^
EMIm-OAc, **2**, H_2_O_2_ (aq. 30%, 1 eq.)	46	33
EMIm-Cl, **2**, H_2_O_2_ (aq. 30%, 1 eq.)	54	27
BMIm-OAc, **2**, H_2_O_2_ (aq. 30%, 1 eq.)	33	35
BMIm-Cl, **2**, H_2_O_2_ (aq. 30%, 1 eq.)	48	32
EMIm-OAc, **2**, H_2_O_2_*urea (1 eq.)	82	8
EMIm-Cl, **2**, H_2_O_2_*urea (1 eq.)	84	5
BMIm-OAc, **2**, H_2_O_2_*urea (1 eq.)	74	13
BMIm-Cl, **2**, H_2_O_2_*urea (1 eq.)	82	11
EMIm-OAc, **2**, H_2_O_2_*urea (2 eq.)	83	17
EMIm-Cl, **2**, H_2_O_2_*urea (2 eq.)	88	12
BMIm-OAc, **2**, H_2_O_2_*urea (2 eq.)	74	26
BMIm-Cl, **2**, H_2_O_2_*urea (2 eq.)	82	18
EMIm-Cl, **2**, H_2_O_2_*urea (2 eq.), alox ^3^ (10 eq.)	92	8
EMIm-Cl, **2**, H_2_O_2_*urea (2 eq.), alox (30 eq.)	98	2
EMIm-Cl, **2**, H_2_O_2_*urea (2 eq.), alox (50 eq.)	quant.	0
BMIm-Cl, **2**, H_2_O_2_*urea (2 eq.), alox (10 eq.)	85	15
EMIm-Cl, **3**, H_2_O_2_*urea (2 eq.), alox (50 eq.)	92	8
BMIm-Cl, **3**, H_2_O_2_*urea (2 eq.), alox (50 eq.)	quant.	0
Dimcarb, **2**, H_2_O_2_*urea (2 eq.), alox (50 eq.)	quant.	0
Dimcarb, **3**, H_2_O_2_*urea (2 eq.), alox (50 eq.)	quant.	0

^1^ reaction at ambient conditions (room temperature and air atmosphere). ^2^ work-up for EMIm/BMIM IL solvent: filtration to remove solids, extraction with n-heptane and GC and analysis of the washed (3 x water) and dried (MgSO_4_) extracts; work-up for dimcarb solvent: see experimental section, direct GC/NMR analysis. ^3^ alox: neutral aluminum oxide, Brockmann grade 1, approx. 50 eq. (molar) rel. to starting amine.

## Data Availability

No data are available beyond those in the published Materials and Methods section.
